# Clinical presentation and in-hospital prognosis of lung cancer patients presenting with suspected and confirmed COVID-19

**DOI:** 10.1590/1414-431X2022e12140

**Published:** 2022-09-12

**Authors:** D. Peixoto, J.P.B. Callia, M.S. Bittencourt, G. Generoso, V.M. Anastácio, J.L. Alves-Jr., T.L. da Silva, J.C. Belizário, B.L.M. Araújo, W. Ho, M.D.P.E. Diz, P.M. Hoff, E. Abdala, K.Y. Ibrahim

**Affiliations:** 1 Departamento de Oncologia, Instituto do Câncer do Estado de São Paulo, Faculdade de Medicina, Universidade de São Paulo, São Paulo, SP, Brasil; 2 Centro de Pesquisa Clínica e Epidemiológica, Hospital Universitário, Universidade de São Paulo, São Paulo, SP, Brasil

**Keywords:** RT-PCR, COVID-19, Cancer, Confirmed, Lung cancer

## Abstract

We sought to compare the clinical presentation and prognosis of patients with lung cancer and confirmed COVID-19 infection to those with negative RT-PCR SARS-CoV-2 results. We included patients with confirmed lung cancer and suspected COVID-19 who presented to the emergency department. The primary outcome was in-hospital mortality and secondary outcomes included admission to intensive care unit (ICU) or mechanical ventilation. We analyzed the characteristics according to RT-PCR results and primary outcome. We constructed a logistic regression for each RT-PCR result group to find potential predictors of the primary outcome. Among 110 individuals with confirmed lung cancer (65±9 years, 51% male), 38 patients had positive RT-PCR and 72 patients had negative RT-PCR. There was no difference between groups for any clinical characteristic or comorbidities though individuals with confirmed COVID-19 had higher functionality in the ECOG scale. Leucocytes and lymphocytes were lower in individuals with positive tests. The primary outcome occurred in 58 (53%) individuals, 37 (34%) were admitted to the ICU, and 29 (26%) required mechanical ventilation. Although mortality was similar between the two groups, individuals with confirmed COVID-19 were significantly more likely to be admitted to the ICU or receive mechanical ventilation. Only lower lymphocytes and higher CRP were significantly associated with higher mortality. The clinical presentation of COVID-19 in lung cancer is not sufficient to identify higher or lower probability groups among symptomatic individuals, the overall mortality is high irrespective of RT-PCR results, and lymphopenia on admission was associated with the diagnosis and prognosis for COVID-19.

## Introduction

Coronavirus disease 2019 (COVID-19), a respiratory tract infection caused by SARS-CoV-2, emerged in Wuhan, China, in December 2019 (1). The virus spread rapidly and affected numerous countries (2). In Brazil, the first case was registered in late February 2020 and its dramatic spread resulted in Brazil having the second highest incidence and mortality in the world (3).

COVID-19 can be associated with severe illness, particularly in high-risk individuals such as cancer patients. Reports on patients with cancer and COVID-19 have suggested a high mortality rate compared with the general population (4,5), as well as more rapid progression to severe disease in patients with malignant tumors than in noncancer patients (5,6).

Although all types of malignancies appear to be associated with high COVID-19 prevalence, morbidity, and mortality, lung cancer represents a specific situation of cumulative risk factors for COVID-19 complications (7,8), including older age, smoking habits, and pre-existing cardiopulmonary comorbidities, in addition to cancer treatment (9,10).

Moreover, clinical practice can be challenging in patients with lung cancer and suspected infection with SARS-CoV-2. First, this group might have prior lung lesions from the cancer itself or from therapy (11). Second, COVID-19 clinical presentation in lung cancer patients can range from an asymptomatic condition to severe respiratory complications requiring intensive care (8,12,13). In the present study, we sought to compare the clinical presentation of patients with lung cancer and confirmed COVID-19 to patients with suspected COVID-19 with negative RT-PCR SARS-CoV-2 results and then to describe the prognosis and predictors of mortality according to these groups.

## Material and Methods

### Ethics approval

This retrospective study was approved by the Institutional Review Board (protocol 1737/20) of the State of São Paulo Cancer Institute (ICESP), which waived informed consent.

### Study design and participants

This cohort study included adult patients (≥18 years of age) from the ICESP, which has 600 beds. We included all patients who presented to the emergency department from March 15 to June 20, 2020, with a confirmed histopathological diagnosis of lung cancer and suspected COVID-19, defined by the presence of at least one of the following symptoms: fever higher than 37.8°C, cough, sore throat, rhinorrhea, dyspnea, anosmia, dysgeusia, oxygen saturation <93%, or respiratory rate >24 breaths per minute, based on WHO criteria (3).

We reviewed the electronic medical records to collect demographic, clinical and laboratory characteristics, as well as treatment and outcome data using a standardized questionnaire of all patients with clinical criteria and RT-PCR SARS-CoV-2 (RT-PCR) test results. Additional details were manually reviewed in the medical history. Asymptomatic patients with RT-PCR collected for other protocols at the hospital were not included in this study.

We collected information on symptoms and signs on admission and also age, sex, cancer histological type, tumor staging, immunosuppressive regimen treatment, comorbidities, smoking status (current, former, or never smoker), Eastern Cooperative Oncology Group (ECOG) performance status (14), hospital and intensive care unit (ICU) admission, mechanical ventilation, and mortality.

The histological type of the cancer was divided in two groups: i) small cell carcinoma; ii) non-small cell carcinoma, which included: adenocarcinoma, squamous cell carcinoma, and giant cell carcinoma. The tumor staging followed the AJCC Cancer Staging Manual from the American Joint Committee on Cancer. Due to the limited sample size, we grouped stages I, II, and III into one group and stage IV (metastasis) into another group. The ECOG was examined on the last outpatient visit prior to admission and was stratified into two categories: scores 1 and 2 in one group and scores 3 and 4 in another.

Anticancer therapy was defined as either cytotoxic chemotherapy or all other therapies such as targeted drugs, endocrine therapy, and immunotherapy given within 30 days prior to admission. Radiotherapy or surgery were also analyzed if given within 30 days prior to admission.

We defined palliative care as present if the patient was previously followed by the specialized palliative care team as an outpatient or had received this diagnosis prior to the COVID-19 investigation.

Clinical variables such as chronic obstructive pulmonary disease (COPD), diabetes mellitus, and hypertension were obtained from clinical records. We stratified smoking status as current smokers, which included those who stopped smoking up to one month prior to admission, and former smokers as those who stopped smoking more than one month before the COVID-19 investigation.

Laboratory data were collected preferably on the day RT-PCR was collected. If no blood tests were obtained on the date of the SARS-CoV-2 test, we used the value of the day before or the day after.

Molecular test results were obtained from institutional databases. A COVID-19 confirmed case was defined as a positive RT-PCR from a respiratory specimen such as oral and nasopharyngeal swab or endotracheal aspirate. After two negative RT-PCRs at least 48 h apart, the patient was classified in the non-COVID group.

### Outcomes

The primary outcome was in-hospital all-cause mortality and secondary outcomes included admission to ICU or mechanical ventilation use.

### Statistical analysis

Continuous results are reported as mean and standard deviations or median and quartile ranges, as appropriate. Normality was evaluated by visual inspection of histograms. Categorical variables are presented as counts and percentages. Comparisons between groups (by RT-PCR result and by primary outcome) were performed with a *t*-test for continuous variables and Fisher's exact test for categorical variables. In addition, bivariate logistic regression analyses were conducted to evaluate potential predictors of all-cause mortality, stratified by RT-PCR result. Additionally, we further evaluated these associations after adjusting for sex and age for predictors that were significant in the univariate analysis, although such analysis should be interpreted with caution given the limited number of events in each group. All analyses was performed using Stata 14 (StataCorp, USA), and a P-value <0.05 was considered significant.

## Results

We included 110 individuals with confirmed lung cancer (65±9 years, 51% male), of which 38 patients had positive RT-PCR and 72 patients had negative RT-PCR. Among them, 101 (92%) had non-small cell carcinoma, 66 (62%) had stage IV (metastasis), and 38 (35%) were on palliative care prior to the admission. The ECOG classification was ≥3 in 36 (33%) patients. Additionally, 45 (41%) patients received anticancer therapy up to 30 days prior to admission, 19 (17%) received radiotherapy, and no patient underwent cancer surgery in the previous 30 days. The mean time from oncological diagnosis and RT-PCR was 223 days (126-604 days). The median duration of symptoms prior to RT-PCR was 3 days (P=0.54). Details of clinical presentations are presented in Table 1.

**Table 1:** Patient characteristics according to RT-PCR SARS-CoV2 results.

Patient characteristics	Total (n=110)	RT-PCR positive (n=38)	RT-PCR negative (n=72)	P value
Age (years)	65±9	65±9	65±9	0.92
Male (n, %)	56 (51%)	20 (53%)	36 (50%)	0.73
Histopathology (n, %)				0.17
Small cell carcinoma	9 (8%)	5 (13%)	6 (6%)	
Non-small cell carcinoma	101 (92%)	33 (87%)	68 (94%)	
Stage (N=106)				0.31
I, II, and III	40 (38%)	16 (44%)	24 (34%)	
IV	66 (62%)	20 (56%)	46 (66%)	
Time from oncological diagnosis to COVID-19 RT-PCR (days)	223 (126-604)	215 (138-557)	224 (126-628)	0.72
ECOG ≥3 (n, %)	36 (33%)	4 (11%)	32 (44%)	<0.01
Palliative care prior to hospital admission (n, %)	38 (35%)	9 (24%)	29 (40%)	0.08
Anticancer therapy in prior 30 days (n, %)	45 (41%)	16 (42%)	29 (40%)	0.85
Radiotherapy in prior 30 days (n, %)	19(17%)	9 (24%)	10 (14%)	0.20
Comorbidities (n, %)				
COPD	36 (33%)	13 (34%)	23 (32%)	0.81
Diabetes mellitus	41 (37%)	16 (42%)	25 (35%)	0.45
Hypertension	57 (52%)	22 (58%)	35 (49%)	0.35
Smoking (n, %)				0.15
Current	23 (21%)	4 (11%)	19 (26%)	
Former	64 (58%)	25 (66%)	39 (54%)	
Time from symptoms to RT-PCR (days)	3 (1-5)	3 (1-4)	3 (1-6)	0.54
Clinical presentation (n, %)				
Cough	58 (53%)	19 (50%)	39 (54%)	0.68
Myalgia	13 (12%)	5 (13%)	8 (11%)	0.75
Headache	8 (7%)	4 (11%)	4 (6%)	0.44
Fever	25 (23%)	11 (29%)	14 (19%)	0.26
Sore throat	3 (3%)	1 (3%)	2 (3%)	1.00
Coryza	10 (9%)	3 (8%)	7 (10%)	1.00
Anosmia	3 (3%)	1 (3%)	2 (3%)	1.00
Chest pain	12 (11%)	4 (11%)	8 (11%)	1.00
GI symptoms (nausea or vomiting or diarrhea)	11 (10%)	2 (5%)	9 (13%)	0.23
Dyspnea	38 (35%)	16 (42%)	22 (31%)	0.23
Laboratory presentation				
Leukocytes (/mm^3^)	9500 (6300-13700)	8000 (4000-12700)	10800 (7300-14700)	0.03
Lymphocytes (/mm^3^)	900 (600-1400)	600 (400-1000)	1000 (700-1500)	0.004
Platelets (/mm^3^)	249000 (166000-331000)	199000 (154000-281000)	272000 (166000-349000)	0.05
C-reactive protein (mg/dL)	119 (63-199)	99 (70-166)	121 (59-209)	0.55
Saturated O_2_ initial presentation (%)	92 (86-95)	92 (86-96)	92 (86-95)	0.62
Respiratory rate initial presentation (ipm)	19 (18-22)	19 (18-22)	19 (18-24)	0.55

Data are reported as mean and standard deviations or median and quartile ranges. *t*-test or Fisher's exact test. ECOG: Eastern Cooperative Oncology Group performance status; GI: gastrointestinal; ipm: inhalations per minute.

Among classic symptoms, 25 (23%) patients presented with fever, 58 (53%) with cough, and 38 (35%) with dyspnea, while anosmia was only present in 3 (3%) patients. Gastrointestinal symptoms (nausea, vomiting, or diarrhea) occurred in 11 (10%) patients. The clinical features of these patients and the comparison between RT-PCR-positive and RT-PCR-negative groups are detailed in Table 1. There was no difference in symptoms between individuals with RT-PCR-positive and -negative tests. There was also no difference between RT-PCR-positive and -negative groups according to smoking status, COPD, diabetes mellitus, or hypertension. While most laboratory findings did not discriminate between the two groups, leucocytes (P=0.03) and lymphocytes (P=0.004) were significantly lower in individuals with RT-PCR positive tests (Table 1).

**Figure 1:**
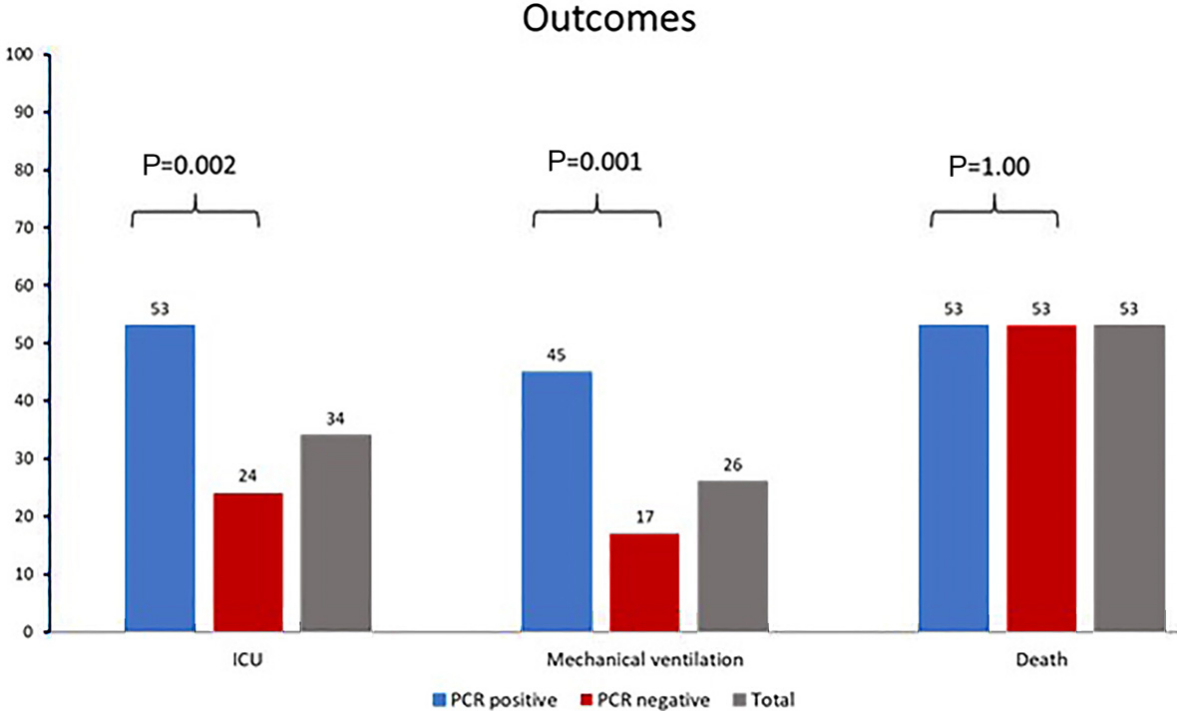
Percent of admissions to intensive care unit (ICU), mechanical ventilation, and deaths, according to RT-PCR SARS-CoV2 status. Fisher's exact test.

All-cause in-hospital mortality occurred in 58 (53%) individuals. A total of 102 (93%) patients were admitted to the hospital, 37 (34%) were admitted to the ICU, and 29 (26%) required mechanical ventilation. While there was no difference in all-cause in-hospital mortality across groups, individuals with RT-PCR positive results were more likely to undergo mechanical ventilation (P=0.001) and be admitted to the ICU (P=0.002) (Figure 1).

Time from oncological diagnosis to RT-PCR was shorter (P=0.005) in individuals who died. However, none of the other clinical characteristics were associated with the primary outcome (Table 2). From the laboratory data, higher leucocytes (P=0.03), lower lymphocytes (P=0.004), and higher C-reactive protein (CRP) (P<0.001) were significantly associated with death (P=0.04).

**Table 2:** Clinical characteristic according to death status.

Patient characteristics	Dead (N=58) 53%	Alive (N=52) 47%	P value
Age (years)	67±8	63±10	0.08
Male (n, %)	32 (55%)	24 (46%)	0.34
Histopathology			0.86
Small cell carcinoma	5 (9%)	4 (8%)	
Non-small cell carcinoma	53 (91%)	48 (92%)	
Stage (N=106)			0.04
I, II, and III	26 (29%)	24 (48%)	
IV	40 (71%)	26 (52%)	
Time from oncological diagnosis to COVID-19 RT-PCR (days)	177 (59-415)	285 (176-729)	0.005
ECOG ≥3 (n, %)	23 (40%)	13 (25%)	0.10
Palliative care prior to hospital admission (n, %)	23 (40%)	15 (29%)	0.23
Anticancer therapy in prior 30 days (n, %)	22 (38%)	23 (44%)	0.50
Radiotherapy in prior 30 days (n, %)	5 (14%)	11 (21%)	0.31
Comorbidities (n, %)			
COPD	19 (33%)	17 (33%)	1.00
Diabetes mellitus	22 (38%)	19 (37%)	1.00
Hypertension	28 (48%)	29 (56%)	0.43
Smoking (n, %)			0.85
Current	11 (19%)	12 (23%)	
Former	35 (60%)	29 (56%)	
Time of symptoms to RT-PCR (days)	2 (1-4)	4 (2-7)	0.06
Clinical presentation (n, %)			
Cough	28 (48%)	30 (58%)	0.32
Dyspnea	18 (31%)	20 (38%)	0.41
Laboratory presentation			
Leukocytes (/mm^3^)	11100 (7000-15500)	8100 (5700-12300)	0.03
Lymphocytes (/mm^3^)	750 (500-1100)	1100 (600-1400)	0.004
Platelets (/mm^3^)	255000 (159000-352000)	249000 (184000-302000)	1.00
C-reactive protein (mg/L)	153 (79-228)	87 (28-136)	<0.001

Data are reported as mean and standard deviations or median and quartile ranges. *t*-test or Fisher's exact test. ECOG: Eastern Cooperative Oncology Group performance status; GI: gastrointestinal.

Of the patients who underwent chest imaging (n=58, 20 of whom with confirmed COVID-19), 75% had positive RT-PCR with typical findings in computed tomography (n=9/12) and 39% had indeterminate findings (n=9/14), while only 9% (n=2/21) of patients with atypical or no pulmonary changes had positive RT-PCR.

When evaluating only patients with positive RT-PCR (n=38), predictors of death were lower lymphocytes (P=0.05) and higher CRP (P=0.01) (Table 3). For those with negative RT-PCR (n=72), an ECOG ≥3 (P=0.05) and higher CRP (P=0.02) were associated with increased odds of dying. In this group, lymphocytes were not associated with death (Table 3). Those results remained essentially unchanged after adjustment for sex and age for the RT-PCR-positive groups. For the first *vs* the other quartiles of lymphocytes, the odds ratio was 4.4 (P=0.05), while the odds ratio for CRP >100 mg/L was still 7.3 (P=0.01) *vs* those with lower CRP levels. For those with negative RT-PCR, the results for lymphocytes remained non-significant after adjustment with an odds ratio of 0.78 (P=0.69), as well as for CRP >100 *vs* <100, with an odds ratio of 2.4 (P=0.08).

**Table 3:** Potential predictors of all-cause mortality according to SARS-CoV-2 RT-PCR results.

	RT-PCR positive (n=38)		RT-PCR negative (n=72)	
	Odds ratio	P-value	Odds ratio	P-value
Age (per 10 years)	1.35	0.41	1.55	0.12
Male	1.22	0.76	1.56	0.35
Small cell (*vs* other)	1.41	0.73	0.89	0.91
Stage IV (*vs* other)	5.13	**0.02**	1.53	0.40
Time from oncological diagnosis to RT-PCR (days)	0.34	0.11	0.45	0.10
Palliative care	2.14	0.34	1.48	0.42
ECOG >2	0.89	0.91	2.58	**0.05**
Anticancer therapy	2.0	0.30	0.46	0.11
Radiotherapy	1.17	0.84	0.33	0.13
COPD	0.67	0.56	1.25	0.66
T2D	0.53	0.35	1.56	0.37
Hypertension	0.78	0.70	0.72	0.49
Cough	0.42	0.20	0.87	0.78
Dyspnea	0.83	0.78	0.65	0.41
Leukocytes (4th *vs* other quartiles)	1.0	1.0	3.20	**0.03**
Lymphocytes (1st *vs* other quartiles)	3.9	**0.05**	0.87	0.80
Platelets (1st *vs* other quartiles)	0.65	0.57	1.78	0.29
CRP (>100 *vs* <100 mg/L)	7.3	**0.01**	3.1	**0.02**

ECOG: Eastern Cooperative Oncology Group performance status; COPD: chronic obstructive pulmonary disease; T2D: type 2 diabetes; CRP: c-reactive protein. Statistically significant P-values are shown in bold type.

## Discussion

In the present study, we demonstrated that in individuals with lung cancer presenting with acute respiratory symptoms, it was not possible to differentiate those with an RT-PCR positive or negative result based on clinical profile. Our study also demonstrated the exceedingly high risk of dying of this population, with mortality above 50% irrespective of RT-PCR results. While high CRP was associated with death in both groups, lower lymphocyte levels were associated with death only in those with positive RT-PCR results. On the other hand, ECOG ≥3, worsening baseline dyspnea, and higher leucocyte levels were associated with death only in those with negative RT-PCR results.

Although the epidemiological and clinical characteristics of patients with COVID-19 has already been described in literature (1,8), it remains challenging for healthcare workers in primary care, and particularly in emergency settings, to determine which oncologic patients are likely to have COVID-19. Initial reports suggest that patients with cancer are more likely to develop severe COVID-19 than the general population (8,10). This risk of developing COVID-19-related complications may be due to impaired immune function due to the cancer itself, cancer treatment, or both (6,15,16). In addition, there is an increasing debate on potential interactions between coronaviruses and anticancer therapies (6,8). Thus, it is unlikely that SARS-CoV-2 affects all patients with cancer equally (10). Lung cancer has the highest morbidity and mortality among the cancers. Some reports highlight the high proportion of patients with lung cancer with confirmed COVID-19 who develop a severe course (6,8,10,17).

COVID-19 presentation can range from mild symptoms to severe acute respiratory distress syndrome in the general population (18). In our study, there was a lower proportion of fever than in other studies in the general population or in cancer patients (16,19). As in other studies, there was no difference in the clinical presentation between individuals with RT-PCR positive and negative results in our study (12,16).

A high ECOG performance score is known to have a deleterious effect on overall outcomes. For those with negative RT-PCR, higher ECOG was associated with increased risk of death. However, in those with positive RT-PCR, only 4 (11%) patients had ECOG ≥3. One hypothesis is that patients with a lower ECOG score are more likely to be outside and more exposed to virus contamination as they are more functional. Remarkably, we found no association between surgery, radiotherapy, or anticancer therapy and all-cause mortality, as previously described (5,6,8). Furthermore, most patients with ECOG ≥3 had negative RT-PCR result. This may suggest that these patients, who are likely to have more end-of-life complications, presented to the emergency department with signs and symptoms similar to those of COVID-19. Therefore, in a pandemic scenario, COVID-19 must be considered as a differential diagnosis in most cases, resulting in more ECOG≥3 cases in negative RT-PCR than in positive RT-PCR patients.

Although clinical presentation could not distinguish between RT-PCR positive and negative patients, the RT-PCR positive group was more likely to have lower lymphocyte levels and higher risk of death. A similar pattern was described for non-cancer patients (16,20). Ultimately, lower lymphocyte level could be used as an ancillary marker for diagnosis and severity in lung cancer patients, especially in centers where more expensive tests such as d-dimer, interleukin-6, and ferritin are not feasible for routine practice (5).

Our study had a high mortality rate compared to other COVID-19 cancer populations such as 28% in the New York Hospital System (21) and 33% at the TERAVOLT registry report (15). This is probably related to the profile of lung cancer individuals such as late diagnosis, worsening cancer prognosis, and rapid evolution to fatality compared to other cancers (22). Other comorbidities such as COPD, diabetes mellitus, and hypertension, which can be associated with increased risk of death in the general population, did not appear as predictors for poor outcomes in our study (23,24).

Despite the comparable mortality, the RT-PCR positive group had a higher ICU admissions (P=0.002) and a greater need of mechanical ventilation (P=0.001). These findings suggested that clinicians considered this population to have a more acute disease course and to be more likely to benefit from intensive care support, despite the similar mortality observed.

Interestingly, death predictors were not the same in the two groups. In the positive RT-PCR group, death was probably not related to the underlying disease, suggesting that COVID-19 was the main determinant of death, indicated by the low lymphocyte levels. On the other hand, in patients with negative RT-PCR results, a higher ECOG score was a predictor of worse prognosis and likely suggest that in this group the underlying cancer might be a major determinant of death. In addition, in the negative RT-PCR group, higher leukocyte values and high CRP were associated with death. This may suggest that bacterial infection or clinical decompensation was associated with mortality. Ultimately, the data suggested that the determinant of death was different in patients with positive and negative RT-PCR and lung cancer.

The present study must be viewed in the context of its design. First, the sample size was limited, which influenced the ability to make adjustments for several possible confounders. Second, due to the limited sensitivity of RT-PCR, the negative group might include some COVID-19 cases. Also, about a half of the patients did not have imaging data. Finally, this study was performed in confirmed lung cancer patients treated at a tertiary care center, and the current findings might not be applicable to other scenarios.

### Conclusions

To the best of our knowledge, this was the first retrospective cohort study in patients with COVID-19 and lung cancer comparing positive and negative RT-PCR tests for SARS-CoV-2. We demonstrated that the clinical profile is not associated with RT-PCR results. Thus, all acute respiratory symptoms in this population should be interpreted as suspected COVID-19. Our study also demonstrated a high mortality rate, irrespective of RT-PCR results. We found that lymphopenia at admission was related to diagnosis and prognosis of COVID-19.
